# Reproductive axis ageing and fertility in men

**DOI:** 10.1007/s11154-022-09759-0

**Published:** 2022-11-02

**Authors:** Sarah Martins da Silva, Richard A Anderson

**Affiliations:** 1 Reproductive Medicine Research Group, School of Medicine, Ninewells Hospital and Medical School, University of Dundee, DD1 9SY Dundee, UK; 2 MRC Centre for Reproductive Health, The Queen’s Medical Research Institute, University of Edinburgh, 47 Little France Crescent, EH16 4TJ Edinburgh, UK

**Keywords:** Male ageing, Male fertility, Reproductive axis, Male hypogonadism, ART, Spermatogenesis

## Abstract

Compared to women, increasing male age is not accompanied by such marked changes in reproductive function but changes certainly do happen. These include alterations to the hypothalamo-pituitary-testicular axis, with resultant implications for testosterone production and bioavailability as well as spermatogenesis. There is a decline in sexual function as men age, with a dramatic increase in the prevalence of erectile dysfunction after the age of 40, which is a marker for both clinically evident as well as covert coronary artery disease. Despite a quantitative decline in spermatogenesis and reduced fecundability, the male potential for fertility persists throughout adult life, however there are also increasingly recognised alterations in sperm quality and function with significant implications for offspring health. These changes are relevant to both natural and medically assisted conception.

## Introduction

**Fig. 1:**
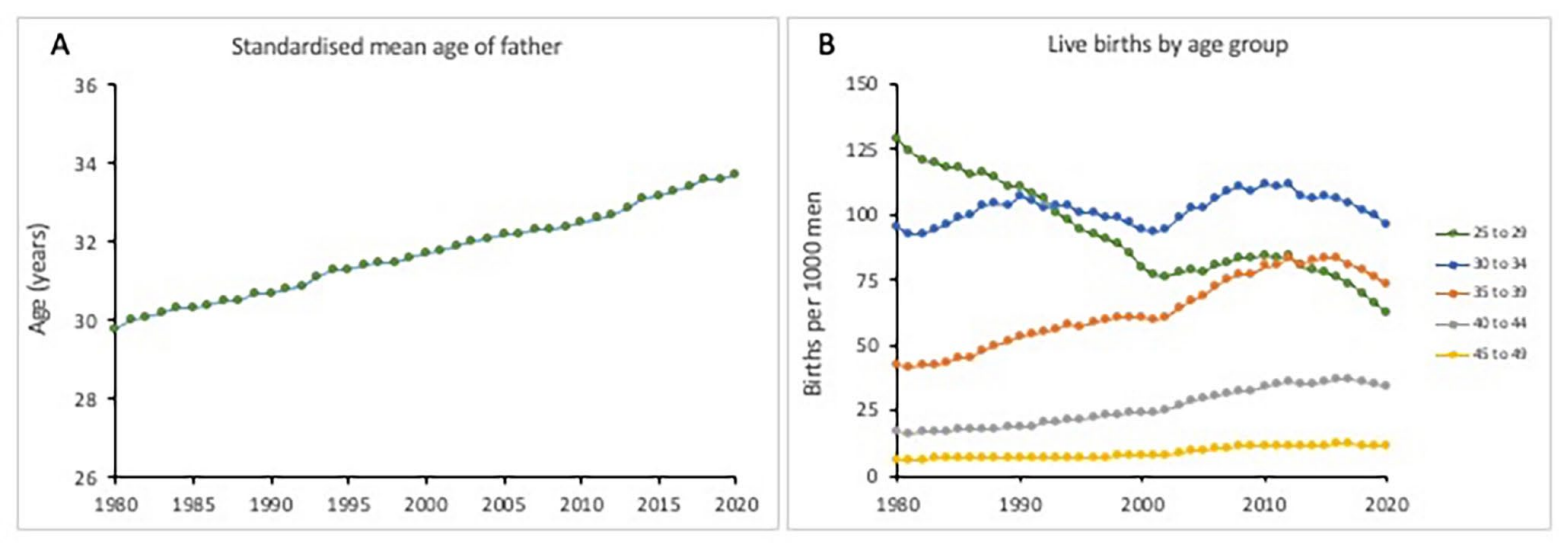
A Standardised mean age at fatherhood in the UK, 1980–2020. B: Age specific fertility rates for men in the UK, 5 year age groups. Data show paternities per 1000 men. Data from The Office of National Statistics (https://www.ons.gov.uk), accessed 5 September 2022

Defined by cellular senescence and telomere attrition, ageing is inevitable and irreversible [1]. The accumulation of genetic, cellular and molecular damage coupled with ineffective repair mechanisms results in a phenotype characterised by decline in function. In men, ageing is accompanied by fundamental changes and dysregulation in the hypothalamic-pituitary-testicular (HPT) axis, with resultant effects on male fertility [2]. However, societal changes mean that men (and women) are increasingly having children at a later age (Fig. 1 A). The average age at fatherhood has risen steadily in the UK over the last four decades, from 29.8 years in 1980 to 33.7 years in 2020 (https://www.ons.gov.uk). Age-specific live birth data show a marked decline in paternity for men in their 20’s and increases in men over 35, although there might be a partial reversal of this over very recent years (Fig. 1B). Whilst the concept of the male biological clock, a term reflecting a decline in male fertility with age, is generally accepted [3], recent studies have challenged traditional beliefs on ageing by suggesting that observed changes reflect age-related alteration in endocrine stimulation rather than ageing of the target organs *per se*. There is certainly evidence that neuroendocrine changes associated with ageing contribute to a decline in reproductive performance in animal models [4]. It is also possible that age-related changes in neuroendocrine function have implications over and above male fertility, for example, potentially deleterious effects on the brain, bone and the cardiovascular system [5]. The concept of male reproductive ageing is therefore now shifting to a model of age-specific alterations in multiple components of the reproductive axis [6].

## Hypothalamic-pituitary-testicular (HPT) axis

The HPT axis represents the anatomic and related endocrine components fundamental to male reproductive function, specifically steroidogenesis and spermatogenesis [7]. The changes in testosterone production with age are detailed in accompanying papers in this series (Matsumoto and Anawalt; Liu), but specific changes relating to fertility are described here. Notably, GnRH pulse frequency and amplitude decreases with age [8, 9], with resultant downstream reduced gonadotrophin secretion and androgen production that characterises late onset hypogonadism. Kisspeptin plays an essential role in the regulation of GnRH secretion [10], pubertal development and reproductive function [11]. In humans, kisspeptin neurons are located in the rostral preoptic area and infundibular nucleus [12]. Kisspeptin acts upstream of GnRH to stimulate the HPT axis by direct signalling to GnRH neurones and pulsatile GnRH release [13, 14]. This is modulated by stimulatory and inhibitory inputs from neurokinin B and dynorphin respectively, which in part are co-localised within kisspeptin-containing neurons (termed KNDy neurons). Hypothalamic levels of kisspeptin (and its receptor G-protein coupled receptor 54; GPR54) decrease with age and are thus likely to contribute to reduced GnRH observed in old age [15]. Peripheral actions of kisspeptin are less well understood although suggested to be related to metabolism, obesity and insulin resistance [16]. Leptin also stimulates the HPT axis, although leptin receptors are not present in GnRH neurons. Given that leptin receptors have been described in kisspeptin neurones [17, 18], it is plausible that changes in this pathway underpin obesity-related leptin resistance of age [19]. Although there is no age-related change in pituitary capacity to secrete gonadotrophins, secretion of luteinising hormone (LH) by the pituitary gland is stimulated by GnRH and thus also declines with age (independently of body weight changes). It is unclear whether diminished testosterone levels associated with male ageing is as a result of fewer Leydig cells or as a result of impaired testosterone production, or both. Age-related decline in number of Leydig cells has been reported using quantitative histometric estimation following autopsy [20], orchidectomy [21] and cadaveric organ donation [22], whereas others report the number of Leydig cells to be relatively unchanged with age [23]. Impaired production of testosterone by Leydig cells in response to LH as an age-related phenomenon has also been reported [24–26]. Emerging data from animal models suggests a possible mechanism of repressed testosterone synthesis via attenuation of circadian clock-related signalling pathways in Leydig cells in response to accumulated endoplasmic reticulum stress with age [27]. Testosterone modulates the secretion of LH through dual feedback to both hypothalamus and pituitary, directly by feedback via interaction with androgen receptors and indirectly following aromatisation of testosterone to oestradiol, acting predominantly at the level of the pituitary gland: overall, testosterone feedback strength also declines with age [26]. Changes in the Sertoli cell product inhibin B and its feedback regulation of FSH have been less investigated, but available data show a decline in inhibin B concentrations with age with a decline in Sertoli cell number [23], and a reciprocal rise in FSH [28], thus analogous to the monotropic rise in FSH in older premenopausal women [29].

As men age, testosterone levels typically fall. From age 30 years, a man’s serum total testosterone levels decline by 1–2% annually. Men > 70 years old tend to have serum testosterone levels that are about one half to two thirds of those of men in their 20s.

In addition, sex hormone–binding globulin (SHBG) levels increase with ageing, causing an even greater decline in serum free and bioavailable testosterone. However, there is ongoing debate regarding how much of this is due purely to ageing, and how much is attributable to co-morbidities, notably obesity [30] with little or no decline seen in very healthy older men [31]. There may also be a secular trend contributing to falling testosterone levels [32, 33]. Nonetheless, health-factor independent age-related longitudinal decline in testosterone and free testosterone index and increase in sex hormone binding globulin (SHBG), was reported in 890 participants from Baltimore Longitudinal Study of Aging (BLSA), America’s longest-running scientific study of human ageing [34]. Notably, a large recent individual patient date (IPD) meta-analysis of data on over 12,000 men aged 25 to 85 years confirms a relatively modest reduction in testosterone levels with increasing age, but with a much more marked decrease in free testosterone, driven by a rise in SHBG [35]. Conversely, an analysis of testosterone levels in 10,097 healthy men from 13 studies showed no decline in testosterone after age 40 but increasing variance [36]. Thus, testosterone deficiency is increasingly common as men age. This is termed late onset hypogonadism (LOH), also known as functional hypogonadism to distinguish from the classical causes of male hypogonadism. Detailed analysis of potentially relevant symptoms indicates that only sexual function symptoms are specific to LOH, with the suggestion that the condition can be defined as biochemical testosterone deficiency (less than 11nmol/l, 3.2ng/ml) in the presence of at least 3 sexual symptoms, i.e. frequency of waking erection, erectile function sufficient for intercourse, and frequency of sexual thoughts [37]. Co-morbidities and obesity are significant contributors to LOH, leading to the suggestion that testosterone may be a biomarker of male health [38]; indeed low testosterone levels are associated with overall mortality [39, 40], although not in all studies [41].

Testosterone is converted to oestradiol by the enzyme aromatase; oestradiol mediates most of testosterone’s action on organs such as bones and the brain. Oestrogen receptors, as well as aromatase, are abundant in the brain (pre-optic area and anterior hypothalamus), penis (vasculature and urothelium) and testes [42]. Oestradiol is essential for modulating libido, erectile function and spermatogenesis, as well as adipose tissue metabolism and regulation. Serum oestradiol concentrations decline with male age in some but not all studies [43], although this is less evident than changes in testosterone, reflecting higher fat mass and increased aromatase activity [44].

## Implications for fertility

Compared to women, men do not show a clear-cut cessation of reproductive capacity, with spermatogenesis persisting into old age. However, decline in testosterone levels may also impact fertility through sexual dysfunction (low libido, erectile dysfunction).

## Libido and sexual function

Libido describes sexual drive or desire for sexual activity and declines as men (and women) age. Decreased testosterone is associated with low libido in men [45]. However, a recent systematic review reported highly variable lack of sexual desire, affecting 12 − 51.6% men aged ≥ 60 years, 20 − 65.9% men aged ≥ 70 years and 40 − 82.4% men aged ≥ 80 years [46]. Almost all men find a normal sex life important as they age and remain sexually active, however, only a minority of elderly men seek help from healthcare professionals or use medication for sexual desire [46]. Men with untreated obstructive sleep apnoea are reported to have high incidence of low libido [47]. The aetiology is likely to be multi-factorial, however, depressed testosterone levels are a common finding.

## Erectile dysfunction

Erectile dysfunction (ED) is the inability to attain or sustain an erection of sufficient quality to permit satisfactory sexual activity. It is the commonest sexual dysfunction in men and predominantly affects men > 40 years of age [48]. Low testosterone and elevated oestradiol increase the incidence of erectile dysfunction independently of each other [42] but other, non-endocrine causes include neurogenic, vascular and iatrogenic [48]. Various estimates indicate around 1:3 men are affected by ED [49, 50], 30 million men to be affected by ED in United States [51] and a global ED prevalence of 3–76.5% [52]. Common to all studies, the incidence of ED increases with male age. The Massachusetts Male Aging Study reported 52% overall prevalence of ED [53], with 40% men affected at age 40 and nearly 70% of men affected at age 70. The prevalence of complete ED also increased from 5% at age 40 to 15% at age 70. Similarly, a recent systematic review reported ED prevalence to increase steeply with age, with 14.3–70% men aged ≥ 60 years, 6.7–48% men aged ≥ 70 years, and 38% men aged ≥ 80 years affected [46].

Importantly, ED is associated with a reduced quality of life for patients and their partners and is also associated with anxiety and depression as well as other co-morbidities. Men with ED have an increased risk of all-cause mortality odds ratio (OR) 1.26 (95% confidence interval (95% CI) 1.01–1.57), as well as CVD mortality OR 1.43 (95% CI 1.00–2.05). Men with ED are 1.33–6.24-times more likely to have benign prostatic hyperplasia (BPH) than men without ED, and 1.68-times more likely to develop dementia than men without ED [52]. The association between ED and cardiovascular disease (CVD) has long been recognized, and studies suggest that ED may be an independent marker of CVD risk. Perhaps more significantly, ED is an early marker for both obstructive and non-obstructive coronary artery disease (CAD) and may reveal the presence of subclinical CAD in otherwise asymptomatic men [54].

## Spermatogenesis and age

In the presence of testosterone, FSH stimulates Sertoli cells and induces spermatogenesis. Spermatogenesis takes 72–74 days and yields about 100 million new spermatozoa each day. However, as men age, there are reduced numbers of Sertoli cells [55] and, as a result, reduced numbers of spermatozoa [56].

A systematic review and meta-analysis evaluated the effect of male age on seven ejaculate traits (semen volume, sperm concentration, total sperm count, morphology, total motility, progressive motility and DNA fragmentation) from almost 94,000 men extracted from 90 studies [57]. The results indicate a consistent age-dependent decline in semen quality. Age was associated with moderate declines in semen volume, total sperm count per ejaculate, total and progressive motility as well as normal morphology. A significant increase in sperm DNA fragmentation was also observed.

Reflecting spermatogenesis, testicular volume also decreases gradually from the age of 60 years onwards [58], with associated higher levels of gonadotrophins and lower bioavailable testosterone. Indeed, a cross-sectional study of testicular volume and inhibin B (a marker of Sertoli cell function and spermatogenesis) in elderly community-dwelling men 75 to 90 years of age showed 31% lower mean testicular volume in men > 75 years of age compared to men aged 18 to 40 years [59]. The difference in testicular volume was accompanied by an almost twofold elevation of serum FSH levels, and 17% lower serum inhibin B, thus a marked decrease in the inhibin B/FSH ratio, suggesting that although Sertoli cell mass is reduced in elderly men, a compensatory increase of FSH stimulation enables Sertoli cell function (and spermatogenesis) to remain relatively intact.

Another study [60] also reported a modest decrease of average testicular size from a maximum of 16.5 ml at 20 to 30 years of age to 14 ml at 80 to 90 years of age. Histological studies on testes of men between young adulthood and old age have also reported clear age-related changes, albeit with large individual variation. The commonest feature of the ageing testis is variable activity ranging from complete spermatogenesis to sclerosis of the seminiferous epithelium. Narrowing of seminiferous tubule diameter, thickening of basal membrane with arrested spermatogenesis and fibrosis, reduced number and vacuolisation of Sertoli cells and multinucleated spermatogenic stem cells are also commonly described. However, some evidence of spermatogenesis is usually preserved even in testes of the oldest men.

A few studies have reported age thresholds for the onset of decline in sperm quality, with the earliest decline starting from the age of 30 to 35 years [61, 62]. A study of infertile males with age groups 20 to 29, 30 to 34, 35 to 39, 40 to 44 and 45 to 55 years with 100 semen samples in each group, identified a significant and linear decrease in sperm motility, morphology and vitality with advancing age. Further analysis of differences in semen parameters between groups and their linear relationship identified age 35 years to be significant [63]. In a study of 11,706 men, a negative correlation was found between age and routine semen parameters: volume, sperm concentration, progressive motility, vitality, total motile spermatozoa and normal-motile spermatozoa, round cell concentration, and hypo-osmotic swelling test values [64]. Computer assisted sperm assessment (CASA) variables were also negatively affected by age. Using age 40 years as a cut-off value, significant differences in most parameters were seen. The same evaluations in a selected subpopulation of men unexposed to known fertility-compromising factors also found a decrease in some parameters with age. Although obesity exerted a significant deleterious effect on older patients’ semen quality, the effects of alcohol consumption and cigarette smoking were mild.

Sperm DNA damage increases with age and is likely to be related to both defective spermatogenesis and increase in oxidative stress (OS). In a retrospective cohort study of 16 945 semen samples from men undertaking infertility evaluation, DNA fragmentation index (DFI, measured using flow cytometry) significantly increased across all age groups. Oxidative stress adducts were lowest in patients < 30 years old and increased as age increased [65]. Another study of 25,445 men reported an association between advancing paternal age and increased sperm DNA damage, with the rate of increase in DNA fragmentation doubling after age 41.6. Using a logistic regression model, the estimated probability of pathological DFI based age alone was 20% for a 40-year-old and a 40% for a 50-year-old man [66]. Nonetheless, DNA fragmentation by age is hugely heterogeneous, thus allowing only generalised assumptions of age in relation to amount of sperm DNA damage.

Another retrospective cohort study of 18,441 semen samples from men up to age 71 years attending an infertility clinic [67] reported a negative correlation between age and semen volume, total sperm count, motility and high DNA stainability (HDS; which indicates sperm with retained histones due to the lack of full protamination), and positive correlation with sperm concentration and DFI. Semen volume and total sperm count declined after 35 years of age, motility and HDS decreased consistently after 30 years of age, whereas sperm concentration and DFI increased from 26 to 30 years of age. DFI was negatively correlated with sperm concentration, total sperm count, motility and normal morphology. Patients aged ≥ 40 years had higher DFI than those aged < 40 years in the entire cohort, in the abnormal semen parameters cohort, and in the normal semen parameters cohort (OR 2.1, 2.0, 1.9, respectively). However, although these studies are large and with consistent findings, the recruitment of men was solely from infertility clinics.

## Natural conception and pregnancy

Given that testicular function and sperm quality deteriorate with age it is not surprising that studies indicate a negative influence of paternal age on fertility and natural conception, and an association between rising male age and delay in conception. However, unlike the female menopause, there is no obvious age threshold for significant deterioration in male fertility. A large population study (Avon Longitudinal Study of pregnancy and childhood; ALSPAC) of pregnant couples in the early 1990’s investigated the effect of paternal age on time to conception. Nine variables were independently related to time to conception, including female age, female BMI, maternal education, maternal and passive smoking, combined oral contraceptive use, years of co-habitation and housing, but after adjustment for these, likelihood of conception within 12 months was significantly lower for older men. Men who took over 12 months to get their partner pregnant were significantly older (32.6 ± 5.9y) than those who achieved a pregnancy within 12 months (30.9 ± 5.4y; P < 0.0001). Compared to men < 25y, adjusted odds ratios for conception within 12 months was 0.62 (95% CI 0.40, 0.98) for men aged 30 to 34 years, 0.50 (95% CI 0.31, 0.81) for men aged 35 to 39 years and 0.51 (95% CI 0.31, 0.86) for men ≥ 40 years [68]. Time to pregnancy (TTP) was also noted to be significantly affected by increase in male age in an observational study of 2112 pregnant women, with a 5-fold increase in TTP for men > 45 years [69]. Similarly, epidemiological studies have reported a significant influence of paternal age on natural conception, with impaired fertility in men older than 35 to 40 years [70]. As might therefore be expected, there is also increasing evidence for cumulative negative effect of combined paternal and maternal age, with female age > 35 years and male age > 40 years identified as significant [71].

Advanced paternal age is also associated with an increased risk of spontaneous miscarriage, although clearly less pronounced than the effect of advanced maternal age [72], as well as other adverse pregnancy outcomes. A study of 12,710 pregnancies from 6979 women using registry data (2011–2015) demonstrated an odds ratio of 2.05 (95% CI 1.06–2.20) of miscarriage where paternal age was ≥ 50 years in comparison with 25 to 29 years of age after adjusting for maternal age, race/ethnicity, socioeconomic status, marital status and pregnancy intention [73]. A recent meta-analysis that included nine studies also showed an increasing risk of miscarriage with advancing paternal age [74]. Significant effects were found in age categories 40 to 44 years (pooled estimate 1.23; 95% CI 1.06–1.43) and ≥ 45 years (1.43; 95% CI 1.13–1.81). A second meta-analysis was performed for the subgroup of studies investigating first trimester miscarriage. This showed similar pooled risk estimates for the first three age categories and a slightly higher pooled risk estimate for age category ≥ 45 years (1.74; 95% CI 1.26–2.41). Recent data also suggest that not only paternal age, but also paternal cardiovascular and metabolic disease are significantly associated with higher risk of pregnancy loss [75].

## Assisted reproduction technology outcomes

The use of large IVF databases allows for investigation of the paternal contribution to all outcomes, and indeed for exploration of mechanisms, such as impaired sperm chromatin integrity and DNA damage. There are however inconsistent findings when considering paternal age and outcomes following fertility treatment, mainly because older men tend to partner older women, which has a much bigger impact than male age per se. Notably, advanced paternal age (APA) continues to lack accepted definition, however, many studies identify age 40 years as significant [76–78]. In keeping with this, age limits for becoming a sperm donor are imposed, at < 46 years in the UK [79], and < 41 years in Canada (https://cfas.ca/_Library/clinical_practice_guidelines/Third-Party-Procreation-AMENDED-.pdf). In the US, the FDA requirements and American Society for Reproductive Medicine recommendations do not set a specific upper age cutoff [80].

APA has been reported to be associated with lower fertilisation rate [81–83], with an estimated reduction of 0.3% ICSI fertilisation rate with each increase in year of male age [84]. APA has also been reported to compromise laboratory key performance indicators (KPIs), for example a negative impact on blastocyst formation, embryo quality as well as clinical outcomes of assisted reproductive technology (ART) [85–88]. A retrospective analysis of 2142 DI cycles in women < 40 years by Koh et al. [89] showed significantly reduced pregnancy rate and longer time to pregnancy in males > 45 years. Mediation analysis to adjust for influence of sperm concentration and motility did not fully account for the effect seen. Examination of male age and female age as continuous and categorical predictors of ART success in a retrospective cohort study of 2425 IVF/ICSI cycles in couples with unexplained infertility found a negative effect of both male and female age on chance of live birth for each additional year of male age, with significantly worse live birth and clinical pregnancy outcomes in men aged > 50 years compared to men < 40 years (P < 0.05) [90]. Similarly, a negative impact on fertilisation rate and clinical pregnancy rate was reported for men > 51 years in a study of 859 IVF and 1632 ICSI cycles [81]. A study of 77,209 nondonor IVF cycles comparing paternal age ≤ 45 years with paternal age ≥ 46 years found a lower likelihood of pregnancy per cycle (adjusted risk ratio (aRR) 0.81; 95% CI 0.76–0.87) and per embryo transfer (aRR 0.85; 95% CI 0.81–0.90). Lower likelihood of live birth per cycle (aRR 0.76; 95% CI 0.72–0.84) and per embryo transfer (aRR 0.82; 95% CI 0.77–0.88) remained after controlling for confounding factors. However, there were no significant differences in live birth or miscarriage where the female partner was < 35 years of age [91]. A single centre retrospective cohort study of 4833 ART cycles involving 4271 men reported also significantly lower probability of live birth with paternal age > 50 years [92]. In a recent meta-analysis, data extracted from 28 studies (16 autologous oocyte studies; 12 donor oocyte studies) were collated to analyse the effect of paternal age at 30–50 years. In autologous ART cycles, paternal age < 40 years resulted in statistically significant higher clinical pregnancy (OR 1.65, 95% CI 1.27–2.15) and live birth rates (OR 2.10, 95% CI 1.25–3.51) as well as reduced miscarriage rate (OR 0.74, 95% CI 0.57–0.94) [93].

Nonetheless, other studies report no impact of paternal age on ART. A large retrospective study comprising 2204 IUI cycles, 1286 IVF cycles and 1412 IVF donor egg cycles examined the role of paternal age on ART outcome and concluded male age to be irrelevant for the range studied [94]. A meta-analysis of 12 studies and 12,538 oocyte donor cycles demonstrated no statistically significant correlation between APA and fertilisation rate, embryo development, biochemical, clinical, clinical pregnancy or live birth rate, although overall quality of evidence was rated as low [95]. A study of 573 single frozen embryo transfers from 473 patients undergoing oocyte donor and autologous IVF cycles demonstrated that paternal age was not associated with pregnancy outcomes after euploid blastocyst transfer [82] and APA alone has been reported to not adversely affect pregnancy, live birth rate or sperm parameters following IUI [96].

Donor oocyte studies overcome maternal age as a confounding factor, but these tend to have small numbers of males with APA, again mostly defined as > 40 years, so it is difficult to extrapolate ART outcomes at ≥ 50 and ≥ 60 years. However, a retrospective cohort analysis of 1023 anonymous egg donation cycles showed significant increase in pregnancy loss, decrease in live birth rate and decrease in blastocyst formation rate with paternal age > 50 years [97]. No significant differences were seen for implantation rate, clinical pregnancy rate or early embryo development to cleavage stage. Luna et al. [98] also found that advanced paternal age negatively impacted on egg donation treatment outcome where paternal age > 60 years. Whilst, there are also contradictory studies that demonstrate no significant association between APA and ART outcomes [99, 100], a recent meta-analysis including 12 donor oocyte studies reported significant increase in number of cleavage-stage embryos (OR 1.67, 95% CI 1.02–2.75) and blastocyst rate (OR 1.61, 95% CI 1.08–2.38) where paternal age was < 50 years, as well as lower miscarriage rate (OR 0.68, 95% CI 0.54–0.86) [93].

In summary, as men age, sperm counts are lower, sperm swimming is poorer, normal morphology and viability are reduced and sperm DNA damage increases. APA lacks a universal definition because deterioration in fertility is progressive and is also influenced by general health and life exposures including obesity. Nonetheless, an increase in time to conception is seen as men age, as well as higher rates of miscarriage and late pregnancy loss. Lower fertilisation rate and poorer embryo development is also seen in the context of assisted conception, with lower clinical pregnancy and live birth rates over 50 years of age.

## APA and offspring health

The impact of advanced female age is dominant in all discussions about fertility, and often used to determine eligibility for state/NHS funding. In contrast, male age is not commonly considered, reflecting the greater ambiguity on its role in reproductive success. However, birth data from developed countries consistently indicates that paternal age is increasing, and links between APA and adverse outcomes of pregnancy and resulting offspring have raised concerns. De novo mutations mainly arise due to errors in replication during spermatogenesis, accumulate in the male germline during ageing and are frequently transmitted to the offspring with deleterious effects [101]. In addition, DNA methylation during spermatogenesis is an active process and susceptible to errors, which are then propagated to subsequent generations. De novo mutations and DNA methylation abnormalities both represent plausible mechanisms for some of the risks that APA poses to offspring health [102]. It is increasingly recognised that higher paternal age is associated with greater risk of specific genetic disorders, as well as a higher risk of congenital birth defects, such as cleft lip/palate, diaphragmatic hernia, urogenital abnormalities and congenital heart disease [103–106]. Higher risk of stillbirth [107, 108], increased risk of childhood cancer [109] and death in childhood [110, 111] and clear links between APA and psychiatric illness including schizophrenia, autism and dyslexia have also been reported [112, 113] with RR of 2.96 for schizophrenia in offspring of men aged > 50 years compared to under 34, independent of classic risk factors for that condition [114, 115]. These studies are derived from national datasets, with thousands, up to millions, of individuals. The significant male age identified in these studies as a time of increased risk is > 45 years old, though importantly this is within a continuum of changing risk, and is not specific to ART.

There are also established associations between APA and offspring risk for certain rare but clinically severe developmental disorders, commonly attributed to genetic mutations, especially de novo single nucleotide variants (dnSNVs), that accumulate with increasing paternal age [116]. These mutations arise in paternal germ cells 3 to 4 times more often than they do in maternal germ cells [117]. APA (> 40 years) is associated with accumulated damage to sperm DNA and defective mitotic and meiotic quality control mechanisms (mismatch repair) during spermatogenesis. This in turn causes both numerical and structural chromosome abnormalities in sperm, including single gene mutations (relative risk (RR) 10), and a significant increase in rare single gene disorders (RR 1.3 to 12) as well as congenital anomalies (RR 1.2) in offspring [118]. APA has been known for a long time to be a feature in the aetiology of achondroplasia [119], and indeed achondroplasia was the first genetic disorder identified to be influenced by paternal age. Achondroplasia is caused by a mutation in fibroblast growth factor receptor 3 (FGFR3) gene and inherited as an autosomal dominant trait. APA is also recognised to be one of the most critically relevant factors in the aetiological of Apert, Crouzon and Pfeiffer syndromes, rare autosomal dominant conditions [120, 121].

The relative effects of advancing male age on multiple genomic defects in human sperm (DFI, HDS/chromatin integrity, gene mutations, and numerical chromosomal abnormalities) were examined by Wyrobek et al. [122]. After adjusting for confounders, they found major associations between age and the frequencies of sperm with DFI and FGFR3 mutations associated with achondroplasia. However, no associations between age and the frequency of sperm with immature chromatin, aneuploidies/diploidies, FGFR2 mutations (Apert syndrome), or sex ratio were found in this cohort. Their findings predicted an increased risk for producing offspring with achondroplasia mutations, a variable risk of fathering offspring with Apert syndrome, but no increased risk for fathering aneuploid offspring (Down, Klinefelter, Turner, triple X, and XYY syndromes) or triploid embryos with advancing paternal age. Despite potential harm to embryo development and offspring, it has been hypothesised that these dominant mutations may become enriched in the male germ line because they confer a selective advantage to the spermatogonial cells in which they arise [123]. A mechanism favouring clonal proliferation of germ cells carrying pathogenic mutations results in increasing numbers of affected sperm as men age, which in turn increases de novo mutations in the offspring of older fathers. This process has been termed “selfish spermatogonial selection” [124]. Epigenetic changes in the sperm of older men have also been reported, but the clinical consequences are unclear [125, 126].

In summary, while genetic defects related to faulty sperm quality control leading to single gene mutations and epigenetic and methylation alterations have been implicated as root causes affecting offspring health, exact mechanistic understanding remains to be clarified [127]. The precise paternal age at which risk develops and the magnitude of the risk are also poorly understood or may have gradual effects [128], although overall, the RR for psychiatric conditions is in the range of 1.5–5.7 and that for single gene disorders is 1.3–12 with paternal age > 40 years [118].

## An endocrine approach to fertility in older men?

Given the reproductive endocrine changes associated with increasing age, notably lower LH levels and reduced Leydig cell responsiveness to LH, it might be possible to reverse or prevent these changes using endocrine therapies. The risks and benefits of replacement of testosterone itself have been fully discussed elsewhere [129] and will not be repeated here, but a key issue is that such treatment will suppress gonadotrophin secretion and thus spermatogenesis. This is indeed the basis of the hormonal approach to male contraception [130], within which context age has been shown to be a modifier of recovery from testosterone treatment, with faster recovery in older men [131]. Testosterone treatment is therefore not appropriate in men who wish to maintain their fertility. Available data on other fertility maintaining approaches in older men are however from mostly small, uncontrolled reports. In young, hypogonadotropic men, LH (in the form of hCG) and FSH are of established value in stimulating testosterone production and spermatogenesis, with induction or restoration of fertility [132]. Anecdotal evidence supports the potential efficacy of this treatment approach in men with idiopathic adult-onset hypogonadism, with restoration of spermatogenesis from azoospermia in some [133].

The oestrogen receptor antagonist clomiphene has also been widely used to try to stimulate testosterone production and spermatogenesis by reducing negative feedback at the hypothalamus and pituitary. A recent meta-analysis of 19 studies including 1642 hypogonadal men indicated efficacy in improving testosterone concentrations (as well as those of LH, FSH, SHBG and oestradiol) and androgen-deficiency symptoms [134]. While most data refer to younger men, stimulation of testosterone levels has been reported in a group of 125 hypogonadal men with mean age 62 years treated with 25 mg clomifene per day for 3 months [135]. Clomiphene comprises a mixture of cis-isomer (zuclomiphene) and trans-isomer (enclomiphene). Of note, enclomiphene has been shown to increase testosterone levels while stimulating FSH and LH production. Published studies suggest that most of the beneficial effects of clomiphene in men with secondary hypogonadism are due to the trans-isomer enclomiphene, whereas zuclomiphene contributes little to the intended outcomes [136]. A comparable alternative approach to reducing feedback inhibition of GnRH secretion is through use of aromatase inhibitors (letrozole and anastrozole), also increasingly widely used in women for ovulation induction [137]. A key difference between these approaches is that estrogen production is prevented by aromatase inhibitors, whereas it increases with clomiphene, thus potentially altering both side-effects and long-term implications, for example for bone mass. Regarding spermatogenesis, the value of this approach for the treatment of infertile men remains unclear [138], but conversely it is clear that spermatogenesis is not suppressed and thus fertility is not adversely impacted. Studies specifically including older men are lacking, but the mechanism of action supports this approach when there is co-existent obesity and increased oestrogen production.

## Addressing the issues of advanced paternal age

The above discussion highlights that there are important specific risks associated with paternity in older men, just as there are for older women. This raises the possibility of interventions, whether purely for provision of information, or even for prevention. Information provision is central to patient understanding and appropriate autonomous decision making, however, this also requires that those giving the information need to be well informed. At present, this may not be the case in the generality of infertility centres, with often very female-orientated approaches to the management of infertility, nor is it likely to be the case in primary care. Improving such knowledge is part of the wider need for greater recognition of the importance of the male contribution to fertility and broader reproductive health [139] and the opportunity for examination, investigation and potentially interventions that engagement of older men in a clinical setting may allow.

At present there are no interventions that can reduce the risks of APA, nor available specific tests that can identify individuals at increased risk. The use of donor sperm (from a younger man) might be a consideration for some couples, but in reality, this is not a common approach and is rarely acceptable. Alternatively, although it is possible to test embryos for specific gene defects e.g. associated with achondroplasia, this is not possible at present without knowledge of the gene mutation to test for, specific to that individual patient. This approach would also require creation of embryos in vitro, either specifically for such testing, or because IVF was otherwise indicated. In either case, the relatively low risk of de novo mutation makes this a very different clinical scenario compared to current practice of genetic testing where prospective parents are known to have a specific genetic abnormality. A further approach would be consideration of sperm storage at a young age. This is analogous to the current and rapidly growing practice of oocyte cryopreservation by women for age-related fertility decline, sometimes known by the rather derogatory term of ‘social’ egg freezing. While having clear theoretic attractions, sperm freezing also attracts significant controversy [140, 141], largely related to the limited chance of later successful use and concerns over commercialisation. Given the much clearer and greater increased risks to female fertility with increasing age, such considerations may well be magnified in relation to sperm cryopreservation for men, particularly given the more limited intervention required to obtain cryostorable gametes. However, cryopreservation of sperm has adverse effects on sperm quality and function [142] and thus a greater requirement for later use of assisted conception treatments. These implications are likely to outweigh the risk of increasing paternal age. Improved education is necessary in relation to age-related changes in female fertility [143]; these challenges may be yet greater for male fertility.

## Conclusions

Reproductive ageing in men is a progressive, often insidious process but nonetheless important to general health as well as fertility. Effects on fertility are increasingly recognised, with the key outcomes being increased risk to offspring rather than inability to conceive. While the chances of an adverse outcome are low in relation to the risks associated with advanced maternal age, they can be of great clinical importance. Increased knowledge and awareness both by prospective fathers and clinicians is presently the main approach, but it is possible that future developments may allow individualised testing and thus personalised advice and even interventions.
